# Supplementary figures and images for: Rapid detection of methicillin-resistant Staphylococcus aureus in positive blood-cultures by recombinase polymerase amplification combined with lateral flow strip
Source: PLoS One. 2022 Jun 30;17(6):e0270686. doi: 10.1371/journal.pone.0270686 (PMC9246191; doi:10.1371/journal.pone.0270686)

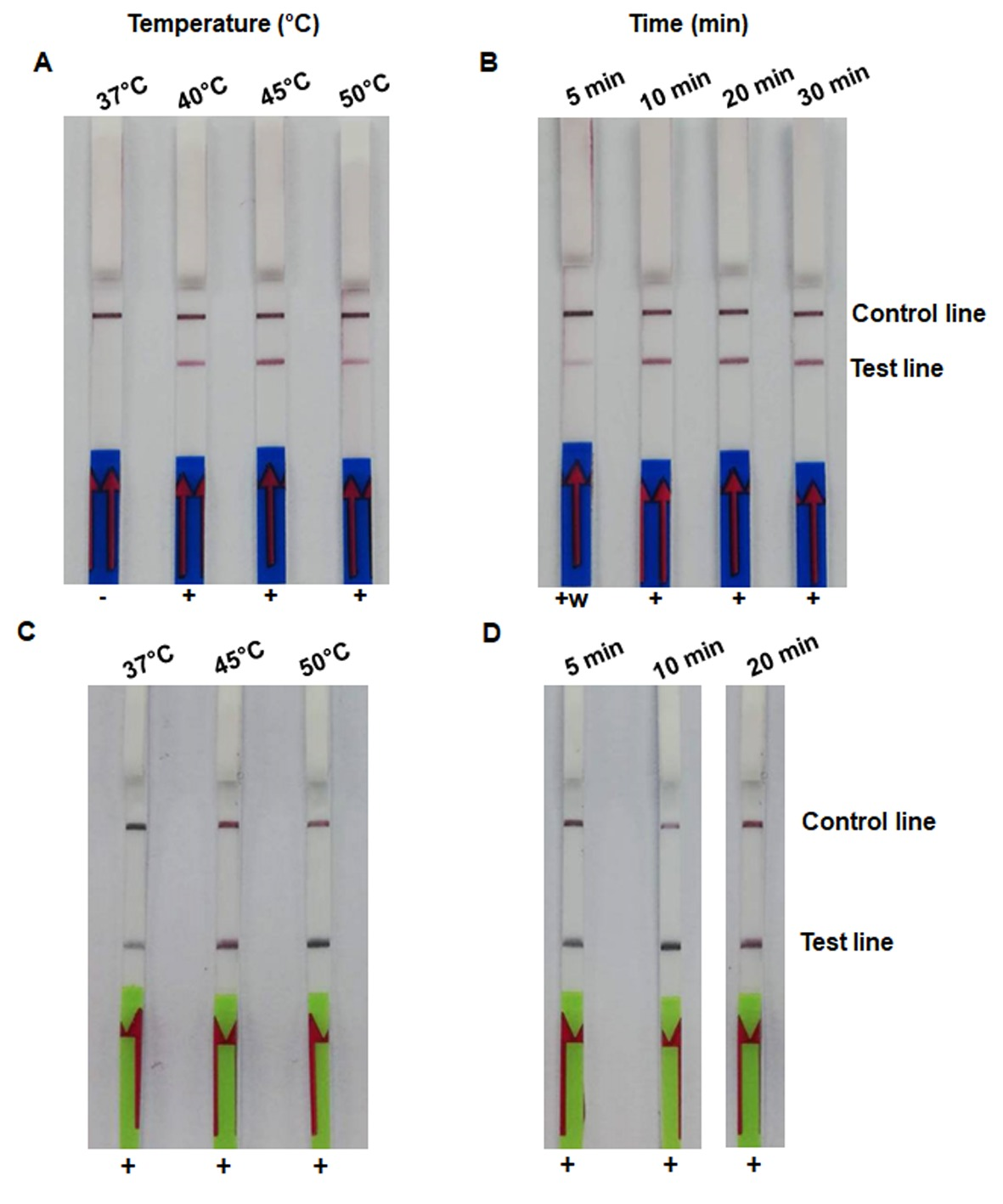

Supplement: S1 Fig — Optimization of incubation temperature and time for nuc- (A, B) and mecA- (C, D) RPA-LF assays. +, positive reaction; +w, weakly positive; -, negative reaction. (TIF) [file pone.0270686.s001.tif]
