# Supplementary material for: Rapid detection of methicillin-resistant Staphylococcus aureus in positive blood-cultures by recombinase polymerase amplification combined with lateral flow strip
Source: PLoS One. 2022 Jun 30;17(6):e0270686. doi: 10.1371/journal.pone.0270686 (PMC9246191; doi:10.1371/journal.pone.0270686)
Supplement: S1 Raw images — (PDF) [file pone.0270686.s002.pdf]

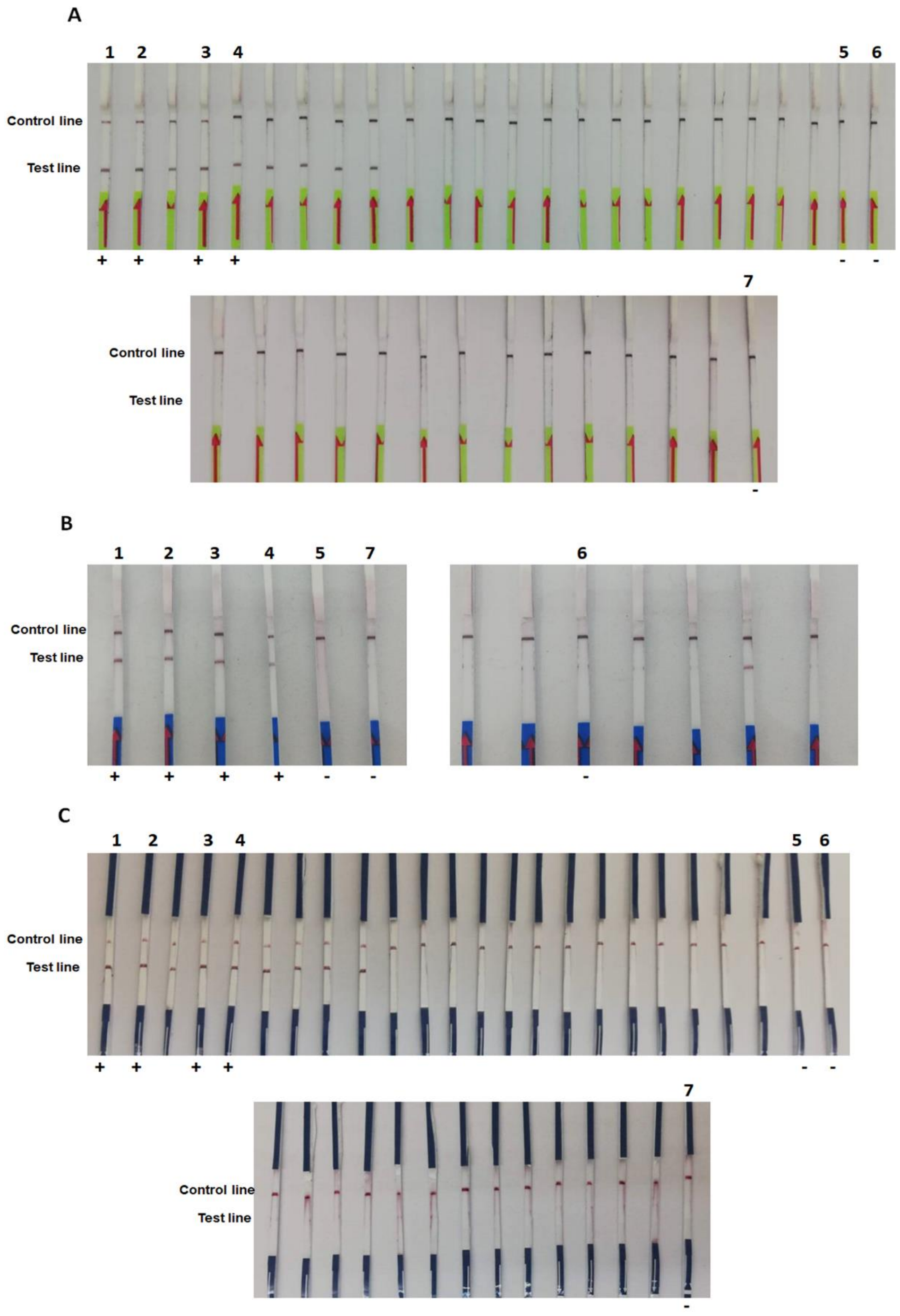

Fig 1 Detection of *nuc*-RPA products by RPA-LF. A, Milenia strips and a Btn-labeled reverse primer; B, Milenia strips and a Dig-labeled primer; C, Kestrel Bio Sciences (KB) strips and a Btn-labeled primer. 1-4, *nuc*-carrying *S. aureus*; 5, non-*nuc*-carrying *Bacillus* spp.; 6, non-*nuc*-carrying *E. coli*; 7, non-*nuc*-carrying *C. neoformans*. +, positive reaction; -, negative reaction.

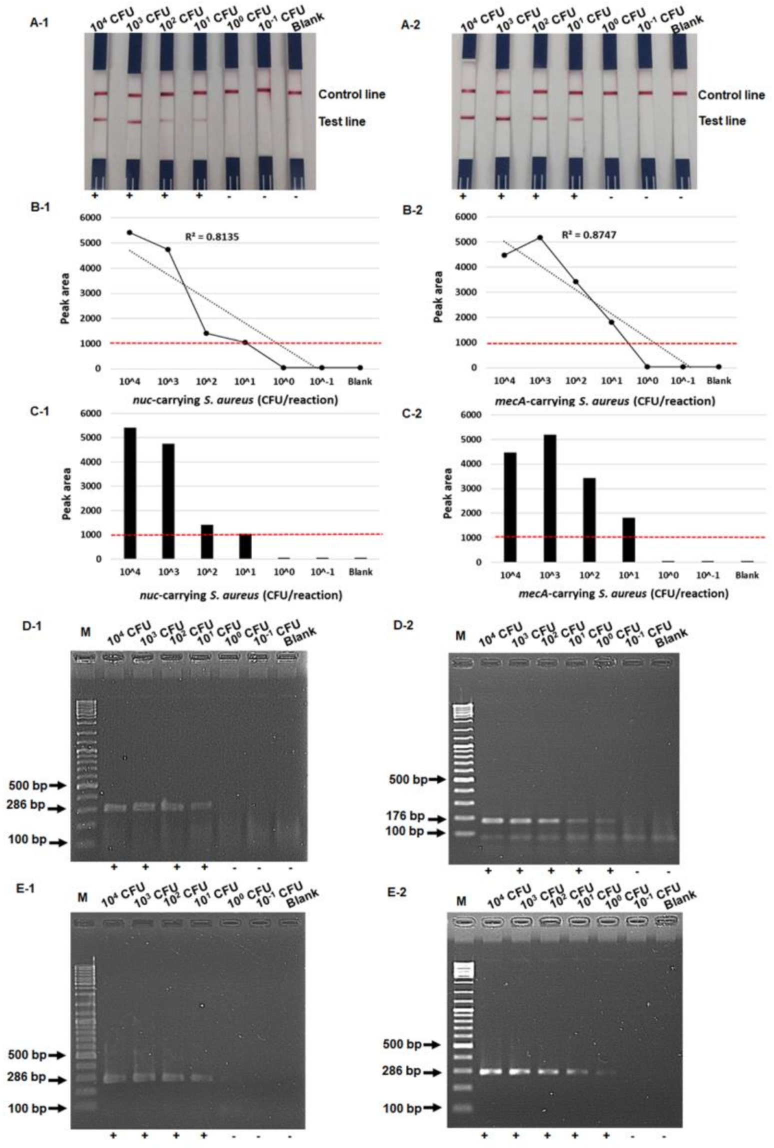

Fig 2 Comparison of the detection limit of the RPA-LF, RPA-AGE and PCR assays. Detection limit of the RPA-LF assay (A) compared with that of the RPA-AGE (D) and PCR (E) assays for detecting *nuc* (1) and *mecA* (2) genes. Lane M, 100 bp DNA ladder. B-1 & B-2, the calibration curve for RPA-LF with different concentrations of *nuc*-carrying *S. aureus* and *mecA*-carrying *S. aureus* corresponding to (A-1 & A-2). C-1 & C-2, Histogram representing peak area of the intensity of the test lines corresponding to (A-1 & A-2). The red dashed lines represent the threshold value (peak area = 1000) above which a result is regarded as positive.

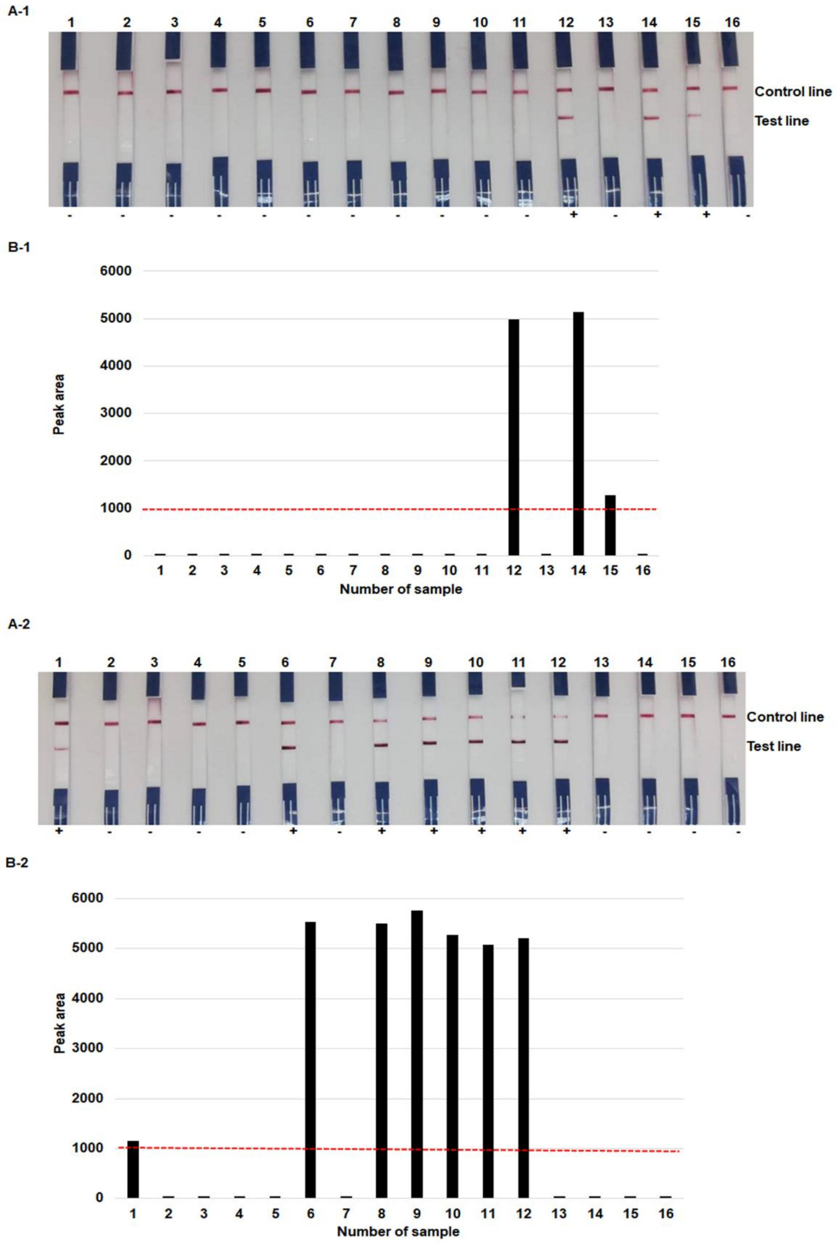

Fig 3 Examples of RPA-LF results for the detection of *nuc* and *mecA* genes. RPA-LF results of *nuc* (1) and *mecA* (2) genes in representative positive blood-culture samples (A) and their corresponding peak area profile plots (B). A-1 & B-1; numbers 1-11, 13 and 16, *nuc* gene-negative samples; 12, 14 and 15, *nuc* gene-positive samples. A-2 & B-2; numbers 2-5, 7, 13-16, *mecA* gene-negative samples; 1, 6, 8-12, *mecA* gene-positive samples. The threshold value of the intensity of peak area to determine a positive/negative result is 1000 (red dashed line).

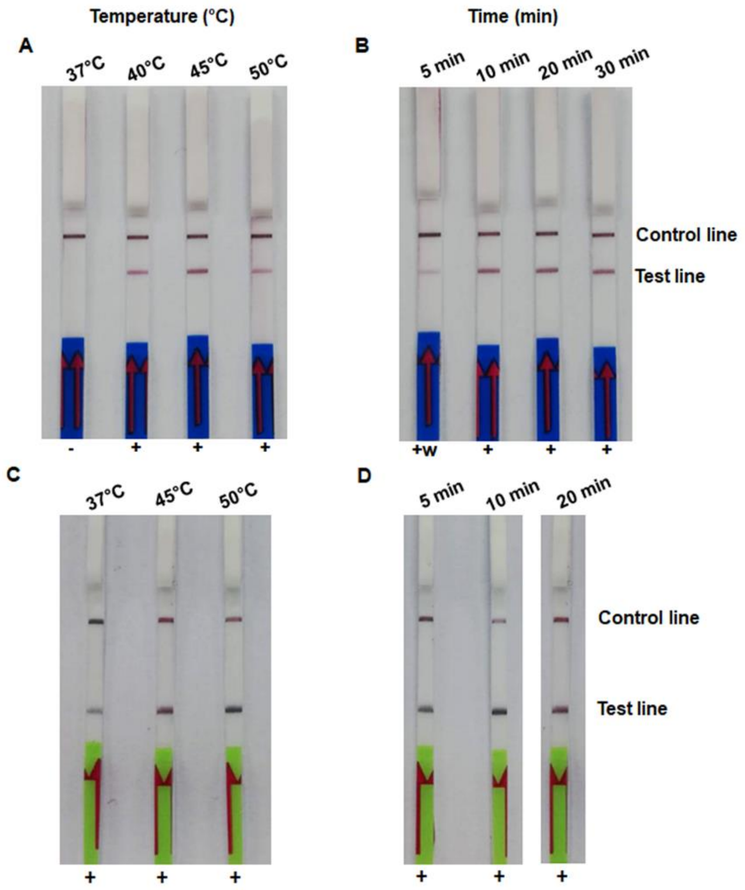

S1 Fig Optimization of incubation temperature and time for nuc- (A, B) and mecA- (C, D) RPA-LF assays. +, positive reaction; +w, weakly positive; -, negative reaction.
