# Supplementary material for: Rapid detection of methicillin-resistant Staphylococcus aureus in positive blood-cultures by recombinase polymerase amplification combined with lateral flow strip
Source: PLoS One. 2022 Jun 30;17(6):e0270686. doi: 10.1371/journal.pone.0270686 (PMC9246191; doi:10.1371/journal.pone.0270686)
Supplement: S1 Table — (PDF) [file pone.0270686.s003.pdf]

S1 Table Diagnostic performance of the RPA-AGE assay in the detection of *nuc* and *mecA* genes in 56 clinical isolate samples

**Pathogens (n)**	**No. of positive isolates by**			
	**PCR**		**RPA-AGE**	
	nuc	***mecA***	***nuc***	***mecA***
***mecA*-carrying *S. aureus* (26)**				
S. aureus (23)	23	23	23	23
S. aureus SCC*mec* I NCTC10442 (1)	1	1	1	1
S. aureus SCC*mec* II (1)	1	1	1	1
S. aureus SCC*mec* III (1)	1	1	1	1
***mecA*-carrying coagulase-negative staphylococci (12)**				
S. sciuri (3)	0	3	0	0
S. haemolyticus SCC*mec* type IX (2)	0	2	0	2
S. saprophyticus (2)	0	2	0	2
S. caprae (1)	0	1	0	1
S. chromogenes (1)	0	1	0	1
S. hyicus (1)	0	1	0	1
S. vitulinus (1)	0	1	0	1
S. xylosus (1)	0	1	0	1
**Non-*mecA*-carrying organisms (18)**				
**coagulase-negative staphylococci (2)**				
S. cohnii spp *urealyticus* (1)	0	0	0	0
S. sciuri (1)	0	0	0	0
***Aerococcus viridans* (1)**	0	0	0	0
**Enterobacterales (5)**				
E. coli (2)	0	0	0	0
Enterobacter spp. (1)	0	0	0	0
K. pneumoniae (2)	0	0	0	0
**Non-fermentative bacteria (5)**				
A. baumannii (3)	0	0	0	0
P. aeruginosa (2)	0	0	0	0
***Enterococcus* spp. (3)**				
E. faecalis (2)	0	0	0	0
E. faecium (1)	0	0	0	0
**Yeast (2)**				
C. albicans (1)	0	0	0	0
C. neoformans (1)	0	0	0	0
**Total (56)**	26	38	26	35
