# Supplementary material for: Rapid detection of methicillin-resistant Staphylococcus aureus in positive blood-cultures by recombinase polymerase amplification combined with lateral flow strip
Source: PLoS One. 2022 Jun 30;17(6):e0270686. doi: 10.1371/journal.pone.0270686 (PMC9246191; doi:10.1371/journal.pone.0270686)
Supplement: S2 Table — (PDF) [file pone.0270686.s004.pdf]

S2 Table Performance of the RPA-LF using Milenia vs. KB strips compared with PCR assay for detecting *nuc* and *mecA* genes in positive blood-cultures from routine Srinagarind Hospital

**Pathogens (n)[a] found in the positive blood culture samples**	**No. of positive samples by**						
	**PCR**		**RPA-LF**				
	nuc	***mecA***	***nuc*-btn[b] (Milenia)**	***nuc*-dig[c] (Milenia)**	***nuc*-btn[b] (KB)**	***mecA*-btn[b] (Milenia)**	***mecA*-btn[b] (KB)**
**Gram-positive bacteria (20)**							
Staphylococcus aureus (11)	11	0	11	11	11	0	0
mecA-carrying *S. aureus* (1)	1	1	1	1	1	1	1
mecA-carrying coagulase-negative staphylococci (5)	0	5	0	0	0	5	5
mecA-carrying *Staphylococcus epidermidis* (1)	0	1	0	0	0	1	1
Micrococcus spp. (1)	0	0	0	0	0	0	0
Bacillus spp. (1)	0	0	0	0	0	0	0
**Gram-negative bacteria (8)**							
Escherichia coli (3)	0	0	0	0	0	0	0
Klebsiella pneumoniae (3)	0	0	0	0	0	0	0
Salmonella group B (1)	0	0	0	0	0	0	0
Salmonella group C (1)	0	0	0	0	0	0	0
**Yeast (2)**							
Cryptococcus neoformans (2)	0	0	0	0	0	0	0
**Total (30)**	12	7	12	12	12	7	7

[a] Pathogens were identified by conventional biochemical tests and/or by the VITEK ®2 automated system

[b] btn, biotin (the reverse primer was labeled with a biotin at the 5′ end to enable LF detection)

[c] dig, digoxin (the reverse primer was labeled with a digoxin at the 5′ end to enable LF detection)

KB, Kestrel Bio Sciences
