# Supplementary material for: Rapid detection of methicillin-resistant Staphylococcus aureus in positive blood-cultures by recombinase polymerase amplification combined with lateral flow strip
Source: PLoS One. 2022 Jun 30;17(6):e0270686. doi: 10.1371/journal.pone.0270686 (PMC9246191; doi:10.1371/journal.pone.0270686)
Supplement: S3 Table — (PDF) [file pone.0270686.s005.pdf]

S3 Table Nucleotide sequences of the RPA primers and probes tested for detection of *nuc* and *mecA* genes used in this study

Primer sets	Primer names	Oligonucleotide sequences (5´ to 3´)	Length (nucleotides)	Expected products (bp)	References
nuc-set 1	nuc-F1	GCATCACAAACAGATAACGGCGT AAATAGAAG	32	139	[13]
	nuc-R1	ACATTAATTTAACCGTATCACCA TCAATCGCT	32		
nuc-set 2	nuc-F2	TTAAGTGCTGGCATATGTATGGC AATCGTTTC	32	164	[20]
	nuc-R2	GCATTTGCTGAGCTACTTAGACT TGAAACTAC	32		
nuc-set 3	nuc-F3(RPA)	GTCTAAGTAGCTCAGCAAATGCA TCACAAACAG	33	141	This study
	nuc-R3(RPA)	CACCATCAATCGCTTTAATTAAT GTCGCAGGTTC	34		
nuc-set 4	nuc-F2	TTAAGTGCTGGCATATGTATGGC AATCGTTTC	32	286	[20]
	nuc-R3(RPA)	CACCATCAATCGCTTTAATTAAT GTCGCAGGTTC	34		This study
	nuc-R3-Dig-RPA-LF	Dig-CACCATCAATCGCTTTAATTAAT GTCGCAGGTTC			This study
	nuc-R3-Btn-RPA-LF	Btn-CACCATCAATCGCTTTAATTAAT GTCGCAGGTTC			This study
	nuc-probe	FAM-CGTAAATAGAAGTGGTTCTGAAG ATCCAAC-[THF]–GTATATAGTGCAACTTC-C3-Spacer			This study
mecA-set 1	mecA-F-(RPA_1)	GCGATAATGGTGAAGTAGAAATG ACTGAACGTCCG	35	176	[21]
	mecA-R-(RPA_1)	TTGAACGTTGCGATCAATGTTAC CGTAGTTTG	32		[18]
	mecA-R-Btn-RPA-LF	Btn-TTGAACGTTGCGATCAATGTTAC CGTAGTTTG			

Primer sets	Primer names	Oligonucleotide sequences (5´ to 3´)	Length (nucleotides)	Expected products (bp)	References
	mecA-probe	FAM-CGTTAAAGATATAAACATTCAGGATCGTAA-[THF]-ATAAAAAAAGTATCTA-C3-Spacer			[18]

Btn, biotin; Dig, digoxin; FAM, Carboxyfluorescein; THF, Tetrahydrofuran; C3 Spacers, a polymerase extension blocking site.
