# Supplementary material for: Rapid detection of methicillin-resistant Staphylococcus aureus in positive blood-cultures by recombinase polymerase amplification combined with lateral flow strip
Source: PLoS One. 2022 Jun 30;17(6):e0270686. doi: 10.1371/journal.pone.0270686 (PMC9246191; doi:10.1371/journal.pone.0270686)
Supplement: S4 Table — (PDF) [file pone.0270686.s006.pdf]

**S4 Table Diagnostic performance of the RPA-LF assay in the detection of *nuc* and *mecA* genes in 60 positive blood samples from Srinagarind Hospital**

| RPA-LF | | Conventional method/PCR | | Sensitivity (%) (95%CI) | Specificity (%) (95%CI) | PPV (%) (95%CI) | NPV (%) (95%CI) |
|---|---|---|---|---|---|---|---|
| | | **Positive** | **Negative** | | | | |
| ***nuc*** | **Positive** | 14 (TP) | 0 (FP) | 93.3 (66.0-99.7) | 100 (90.2-100) | 100 (73.2-100) | 97.8 (87.0-99.9) |
| | **Negative** | 1 (FN) | 45 (TN) | | | | |
| ***mecA*** | **Positive** | 16 (TP) | 1 (FP) | 100 (75.9-100) | 97.7 (86.5-99.9) | 94.1 (69.2-99.7) | 100 (89.8-100) |
| | **Negative** | 0 (FN) | 43 (TN) | | | | |

Sensitivity = [TP/(TP + FN)] × 100, specificity = [TN/(TN + FP)] × 100

TP, true positive; FP, false positive; FN, false negative; TN, true negative; PPV, positive predictive value; NPV, negative predictive value

95% CI, 95% confidence interval
