# Supplementary material for: Rapid detection of methicillin-resistant Staphylococcus aureus in positive blood-cultures by recombinase polymerase amplification combined with lateral flow strip
Source: PLoS One. 2022 Jun 30;17(6):e0270686. doi: 10.1371/journal.pone.0270686 (PMC9246191; doi:10.1371/journal.pone.0270686)
Supplement: Attachment — Submitted filename: Response to Reviewers_PONE-D-22-05183.pdf [file pone.0270686.s008.pdf]

June 1, 2022

Dear Editor,

Manuscript ID: PONE-D-22-05183

Title: Rapid detection of methicillin-resistant *Staphylococcus aureus* in positive blood-cultures by recombinase polymerase amplification combined with lateral flow strip

Thank you very much for valuable comments and for giving us an opportunity to revise our manuscript. We have responses to all the issues raised by the editor and reviewers as in the following and use the "Track Changes" function in Microsoft Word of the revised manuscript. We would be grateful if you would consider our revised manuscript for publication.

Yours sincerely,

Aroonlug Lulitanond

Corresponding author

The response to editor's and reviewer's comments

Journal Requirements:

When submitting your revision, we need you to address these additional requirements.

1. Please ensure that your manuscript meets PLOS ONE's style requirements, including those for file naming. The PLOS ONE style templates can be found at https://journals.plos.org/plosone/s/file?id=wjVg/PLOSOne_formatting_sample_main_body.pdf and https://journals.plos.org/plosone/s/file?id=ba62/PLOSOne_formatting_sample_title_authors_affiliations.pdf

Response: *We have already checked PLOS ONE style as suggested. Our manuscript was prepared accordingly the PLOS ONE style.*

2. PLOS ONE now requires that authors provide the original uncropped and unadjusted images underlying all blot or gel results reported in a submission's figures or Supporting Information files. This policy and the journal's other requirements for blot/gel reporting and figure

preparation are described in detail at https://journals.plos.org/plosone/s/figures#loc-blot-and-gel-reporting-requirements and https://journals.plos.org/plosone/s/figures#loc-preparing-figures-from-image-files. When you submit your revised manuscript, please ensure that your figures adhere fully to these guidelines and provide the original underlying images for all blot or gel data reported in your submission. See the following link for instructions on providing the original image data: https://journals.plos.org/plosone/s/figures#loc-original-images-for-blots-and-gels.

In your cover letter, please note whether your blot/gel image data are in Supporting Information or posted at a public data repository, provide the repository URL if relevant, and provide specific details as to which raw blot/gel images, if any, are not available. Email us at plosone@plos.org if you have any questions

Response: *Our manuscript provided the original images for all gel data reported in our submission. We already checked the policy and the journal's other requirements for blot/gel reporting and figure preparation as suggested. We have confirmed that our figures adhere to the guidelines. Our blot/gel image data are not in Supporting Information or posted at a public data repository.*

3. We note that you have included the phrase "data not shown" in your manuscript. Unfortunately, this does not meet our data sharing requirements. PLOS does not permit references to inaccessible data. We require that authors provide all relevant data within the paper, Supporting Information files, or in an acceptable, public repository. Please add a citation to support this phrase or upload the data that corresponds with these findings to a stable repository (such as Figshare or Dryad) and provide and URLs, DOIs, or accession numbers that may be used to access these data. Or, if the data are not a core part of the research being presented in your study, we ask that you remove the phrase that refers to these data.

Please review your reference list to ensure that it is complete and correct. If you have cited papers that have been retracted, please include the rationale for doing so in the manuscript text, or remove these references and replace them with relevant current references. Any changes to the reference list should be mentioned in the rebuttal letter that accompanies your revised manuscript. If you need to cite a retracted article, indicate the article's retracted status in the References list and also include a citation and full reference for the retraction notice.

Response: *The phrases "data not shown" in our manuscript were removed as suggested. The data are not a core part of the research. We have already checked carefully reference list and confirmed that it is complete and correct.*

Additional Editor Comments (if provided):

1. Manuscript requires corrections as pointed out but by reviewers! Please give justification: as in method section 56 isolates (26 *S. aureus* and 30 non-*S. aureus*) collected but in Table 1, there are total 60 isolates. Please check which one is correct.

Response: *We have already checked as suggested. The 56 isolates (26 S. aureus and 30 non-S. aureus) were used for evaluating the performance of the chosen primer set on the RPA-*

AGE test for detection of MRSA colonies (S1 Table) whereas the 60 samples were clinical blood-culture bottles from a routine laboratory in hospital which were used for evaluating the performance of RPA-LF test (Table 1). So, we have specified this point in lines 86-88, 90-94, and 126-128.

2) why isolates other than *S. aureus* and MRSA are listed and studied? is it relevant to the title of the manuscript? study is related to *S. aureus* and MRSA only?

Response: *The title of the manuscript is related to S. aureus and MRSA only because we want to develop the RPA assay for the detection of nuc and mecA genes to diagnose S. aureus and its methicillin-resistance status. The organisms other than S. aureus and MRSA are included for testing the specificity of the developed RPA method. A positive result must be generated only with S. aureus and MRSA strains, but not with the other organisms. We have added this matter in lines 98-99.*

Reviewers' comments:

Responses to Reviewer's Questions

Comments to the Author

1. Is the manuscript technically sound, and do the data support the conclusions?

The manuscript must describe a technically sound piece of scientific research with data that supports the conclusions. Experiments must have been conducted rigorously, with appropriate controls, replication, and sample sizes. The conclusions must be drawn appropriately based on the data presented.

Reviewer #1: Yes

Reviewer #2: Yes

Response: *Thank you very much.*

---

2. Has the statistical analysis been performed appropriately and rigorously?

Reviewer #1: Yes

Reviewer #2: I Don't Know

Response: *Thank you very much. We have described the sensitivity and specificity (statistical analysis) of RPA assay for the detection of methicillin resistant Staphylococcus aureus.*

3. Have the authors made all data underlying the findings in their manuscript fully available?

The PLOS Data policy requires authors to make all data underlying the findings described in their manuscript fully available without restriction, with rare exception (please refer to the Data Availability Statement in the manuscript PDF file). The data should be provided as part of the manuscript or its supporting information, or deposited to a public repository. For example, in addition to summary statistics, the data points behind means, medians and variance measures should be available. If there are restrictions on publicly sharing data—e.g. participant privacy or use of data from a third party—those must be specified.

Reviewer #1: Yes

Reviewer #2: Yes

Response: *Thank you very much.*

---

4. Is the manuscript presented in an intelligible fashion and written in standard English?

PLOS ONE does not copyedit accepted manuscripts, so the language in submitted articles must be clear, correct, and unambiguous. Any typographical or grammatical errors should be corrected at revision, so please note any specific errors here.

Reviewer #1: Yes

Reviewer #2: Yes

Response: *Thank you very much.*

---

5. Review Comments to the Author

Please use the space provided to explain your answers to the questions above. You may also include additional comments for the author, including concerns about dual publication, research ethics, or publication ethics. (Please upload your review as an attachment if it exceeds 20,000 characters)

Reviewer #1: The authors described the sensitivity and specificity of RPA assay for the detection of methicillin resistant *Staphylococcus aureus*. The manuscript has been well written. It can accepted for publication.

Response: *Thank you very much for accepting our manuscript for publication.*

Reviewer #2: The manuscript is well written and findings are important in rapid detection of MRSA from blood culture. However, some changes are suggested

1) In Material and method section it is mentioned that, 56 isolates (26 *S. aureus* and 30 non-*S. aureus*) collected but in Table 1, there are total 60 isolates. Please check which one is correct.

Response: *We have already checked as suggested. The 56 isolates (26 S. aureus and 30 non-S. aureus) were used for evaluating the performance of the chosen primer set on the RPA-AGE test for detection of MRSA colonies (S1 Table) whereas the 60 samples were clinical blood-culture bottles from a routine laboratory in hospital which were used for evaluating the performance of RPA-LF test (Table 1). So, we have specified this point in lines 86-88, 90-94, and 126-128.*

2) Organism other than *S. aureus* and MRSA are listed. As per the title of the manuscript, study is related to *S. aureus* and MRSA only.

Response: *The title of the manuscript is related to S. aureus and MRSA only because we would like to develop the RPA assay for the detection of nuc and mecA genes to diagnose S. aureus and its methicillin-resistance status. The organisms other than S. aureus and MRSA are included for testing the specificity of the developed RPA method. A positive result must be generated only with S. aureus and MRSA strains, but not with the other organisms. We have added this matter in lines 98-99.*

---

6. PLOS authors have the option to publish the peer review history of their article (what does this mean?). If published, this will include your full peer review and any attached files.

If you choose "no", your identity will remain anonymous but your review may still be made public.

Do you want your identity to be public for this peer review? For information about this choice, including consent withdrawal, please see our Privacy Policy.

Reviewer #1: **Yes:** Megha Raj Banjara

Reviewer #2: **Yes:** Dharm Raj Bhatta

Response: *Thank you very much.*
