# Supplementary material for: Rapid detection of methicillin-resistant Staphylococcus aureus in positive blood-cultures by recombinase polymerase amplification combined with lateral flow strip
Source: PLoS One. 2022 Jun 30;17(6):e0270686. doi: 10.1371/journal.pone.0270686 (PMC9246191; doi:10.1371/journal.pone.0270686)
Supplement: Attachment — Submitted filename: PONE-D-22-05183.docx [file pone.0270686.s007.docx]

Manuscript title: Rapid detection of methicillin-resistant *Staphylococcus aureus* in positive blood-cultures by recombinase polymerase amplification combined with lateral flow strip

Date: 14 April 2022

The authors described the sensitivity and specificity of RPA assay for the detection of methicillin resistant *Staphylococcus aureus*. The manuscript has been well written. It can accepted for publication.
